# Leveraging Integrated Primary Care to Enhance the Health System Response to IPV: Moving toward Primary Prevention Primary Care

**DOI:** 10.3390/ijerph20095701

**Published:** 2023-05-01

**Authors:** Nicole Trabold, Paul R. King, Dev Crasta, Katherine M. Iverson, Cory A. Crane, Katherine Buckheit, Stephen C. Bosco, Jennifer S. Funderburk

**Affiliations:** 1 Center for Integrated Healthcare, Syracuse VA Medical Center, Syracuse, NY 13210, USA; 2 Rochester Institute of Technology, College of Health Science and Technology, Rochester, NY 14623, USA; 3 Center for Integrated Healthcare, VA Western New York Healthcare System, Buffalo, NY 14215, USA; 4 Department of Counseling, School, and Educational Psychology, University at Buffalo, Buffalo, NY 14260, USA; 5 Center of Excellence for Suicide Prevention, Canandaigua VA Medical Center, Canandaigua, NY 14424, USA; 6 Department of Psychiatry, University of Rochester Medical Center, Rochester, NY 14642, USA; 7 Women’s Health Sciences Division of the National Center for PTSD, VA Boston Healthcare System, Boston, MA 02130, USA; 8 Department of Psychiatry, Boston University Chobanian & Avedisian School of Medicine, Boston, MA 02118, USA; 9 Department of Psychology, Syracuse University, Syracuse, NY 13244, USA

**Keywords:** intimate partner violence, screening, interventions, primary prevention, integrated primary care

## Abstract

Intimate partner violence (IPV) is a prominent public health problem in the United States, with significant health impacts that are often severe and persistent. Healthcare systems have been called upon to improve both the systematic identification and treatment of IPV largely by adopting secondary and tertiary prevention efforts. Research to date demonstrates both benefits and challenges with the current strategies employed. In this paper, we summarize current knowledge about the healthcare system’s response to IPV and evaluate the strengths, limitations, and opportunities. We offer recommendations to broaden the continuum of healthcare resources to address IPV, which include a population health approach to primary prevention.

## 1. Introduction

Intimate partner violence (IPV) is a prominent public health problem in the United States (U.S.) that involves the use of physical, sexual, or psychological violence (including coercive acts), financial abuse, and/or stalking behaviors with a current or former intimate partner [1,2]. Despite policy initiatives, criminal justice responses, and interventions developed and deployed over the past 40 years, IPV remains a public health crisis with profound impacts on individuals, families, and communities [3,4,5,6] and is related to increased rates of child abuse, suicide, and community gun violence [7,8,9]. The most recent National Intimate Partner and Sexual Violence Survey (NISVS) reports staggering lifetime rates of IPV, with over 47% of women and 44% of men living in the U.S. reporting some form of aggressive physical contact, sexual violence, or stalking, and over 61 million women and 53 million men reporting psychological aggression, which includes insults, humiliation, entrapment and coercive control [10]. While IPV often happens in a larger context of power and control where one partner is primarily using aggressive, abusive, or violent behaviors, IPV may be bidirectional such that both partners use aggression, abuse, and violence toward each other in self-defense, retaliation, or when both individuals are primary aggressors [11,12]. However, the NISVS report found that women are more likely than men to report severe physical violence, injury, violence-related fear, needing assistance from law enforcement, and numerous adverse outcomes that are often severe and pervasive [13]. Research indicates about 81% of women who experience any form of aggression and/or violence report significant and ongoing repercussions, such as physical injuries (including but not limited to repeated concussions and hypoxic brain injury, heart health risk, and pain), depression, anxiety disorders, post-traumatic stress disorder, and substance use [4,14,15]. Health complications often persist for years, leading to increased contact with healthcare providers and high medical costs [16,17]. Recent estimates of the population economic burden of IPV is $3.6 trillion dollars, $2.1 trillion of which is in medical costs, and the remainder is attributable to lost productivity, criminal justice costs, and property loss/damage [18]. 

## 2. United States Healthcare System 

The U.S. healthcare system is a complex interplay among providers, patients, and government and private insurers/payers that provide care to a population of over 300 million people. Unlike other developed countries, the U.S. does not have a National Health Service or universal healthcare coverage. In the U.S., about 50% of the population receives employment-sponsored private insurance, 34% from a government program (i.e., Medicaid), and about 2% from the military (i.e., Veterans Health Administration (VHA)) [19]. The delivery of healthcare is also complex and multifaceted, and is organized and delivered in various settings such as primary care, outpatient specialty care, acute or hospital care, mental healthcare, and long-term care [20]. 

The U.S. healthcare system has long been driven by the medical model of care focused on treating symptoms and curing disease to restore health. However, the benefits of preventative medicine have generated renewed interest in improving healthcare delivery through the adoption of primary, secondary, and tertiary prevention strategies across various healthcare delivery systems. As summarized in Table 1, these prevention strategies can be defined as (1) primary prevention, which is focused on approaches, programs, and strategies that include education to prevent disease/conditions before they occur (i.e., promoting safe condom use), and is often the focus in primary care settings; (2) secondary prevention, which is focused on the early or asymptomatic detection of disease/conditions (i.e., mammography for early detection of breast cancer), and can be implemented across all healthcare delivery systems; and (3) tertiary prevention, which is commonly seen in specialty, acute, and mental healthcare, and is focused on interrupting disease/condition progression, i.e., stroke rehabilitation and chronic disease management [i.e. stroke rehabilitation, chronic disease management, [21]]. While the U.S. healthcare system has long focused on moving toward the absence of disease, primary prevention approaches have grown in the 20th and 21st centuries, shifting focus from treatment to understanding and preventing disease/conditions with healthy populations. While not ubiquitous across all health concerns, the growth of primary prevention within primary care emerged after the American Medical Association endorsed periodic health examinations to reduce mortality. Subsequent research found evidence of clinical effectiveness and reductions in morbidity and mortality with the adoption of primary prevention strategies across various health conditions [21]. Additional changes to the U.S. healthcare system resulted from the passing of the Affordable Care Act (ACA) in 2010 [22]. The ACA included expansions in healthcare insurance beyond employer-sponsored programs and expanded access to preventive medicine, such as screening and counseling in primary care [23,24]. The ACA sought to strengthen primary care delivery by creating improved financial reimbursement to primary care providers and supporting innovative care delivery models, which included a team-based approach driven by a patient-centered care model to improve health outcomes and contain healthcare costs [25,26]. Team-based models strive to meet patient needs by leveraging the varied expertise of healthcare professionals, including, but not limited to, medical providers, pharmacists, rehabilitation providers (physical, speech), dietitians, and mental health providers [27]. However, the implementation of team-based models has varied widely due to various contextual factors of healthcare settings [26]. In summary, as the landscape of the U.S. healthcare system has shifted, new models of care are developing that are emphasizing team-based, prevention-focused primary care to improve health outcomes and overall population health [28].

## 3. Current U.S. Healthcare Response to IPV

As the U.S. healthcare system evolves, the prominence and impact of IPV have been identified as an important social determinant of health. As such, the U.S. healthcare system has been called upon to improve both the systematic identification and treatment of IPV with the adoption of primary, secondary, and tertiary prevention strategies across various healthcare delivery systems. The last decade has seen the development of national and international guidelines that offer clear concrete guidance for all three forms of prevention at the community level and within healthcare systems (Table 1). Primary prevention has been recommended to reduce IPV incidence across all phases of life, beginning in youth, and across multiple points of service, including within healthcare as well as schools and community agencies [29], but the application of primary prevention strategies within healthcare has been slower to develop. However, the secondary prevention strategy of IPV screening has been endorsed by numerous U.S. medical organizations such as the American College of Physicians (ACP), the American Medical Association (AMA), the American Academy of Family Practitioners (AAFP), and the American College of Obstetrics and Gynecologists (ACOG). Further, the U.S. Preventative Services Task Force, an independent panel of national experts that make evidence-based recommendations on primary, secondary, and tertiary preventive services, all recommend the implementation of routine screening to identify IPV in U.S. healthcare settings [33]. These recommendations are limited to screening for the experience of IPV among women. Tertiary prevention strategies, see [34,35], have also been deployed in numerous healthcare environments as a complement to community-based interventions (i.e., legal interventions such as protective orders; temporary housing) to provide treatment and optimize outcomes for patients already experiencing the physical and emotional consequences of IPV. Of these, preventive efforts have focused heavily on secondary prevention within the primary care setting. The first U.S. Preventive Task Force recommendation on IPV screening was issued in 1996, reporting insufficient evidence to make a recommendation on IPV screening with updated recommendations issued in 2004, 2012, and 2018 with new guidelines currently recommending clinicians screen for IPV in women of reproductive age and provide referrals to ongoing support services [36,37,38].

Primary care (PC) serves as an entry point to healthcare (American Academy of Family Physicians), providing the opportunity for secondary prevention strategies, such as screening for IPV, to be provided to a larger proportion of patients. Though mixed results on the overall utility of screening have been reported in the literature (e.g., due to limited direct improvements to overall health and mental health outcomes following screening [39,40,41]), there are numerous benefits to screening. IPV screening is effective in identifying abuse and violence [42], with computer-assisted, self-administered screening increasing the odds of disclosure by 37% [43]. Women generally approve of being asked about IPV regardless of their personal experience with IPV [44] and have described IPV screening as having an educational effect (i.e., increased awareness) that, over time, builds trust in medical providers, which starts the trajectory of help [44]. Further, IPV screening can serve as a catalyst for referral to treatment [45]. Therefore, routine IPV screening increases awareness, destigmatizes IPV, and builds trust in healthcare providers, which creates opportunities for receiving help [46,47,48].

Yet, implementation of IPV screening varies across U.S. healthcare organizations. Efforts to incorporate standard IPV screening have been seen in primary and specialty care (e.g., obstetrics and gynecology), acute care (e.g., emergency departments), and mental or behavioral health settings [45]. Women tend to be the target screening demographic, but there are no uniformly accepted procedures for implementation, as evidenced by wide-reaching differences in screening tools, frequency of screening, and follow-up response; see [see [45]]. A systematic review of IPV screening in routine care concluded that, among U.S. studies, implementation reach within each study (i.e., the proportion of women receiving screening during the study) was approximately 80% [45].

Some healthcare organizations have instituted standard secondary and tertiary prevention strategies as part of their comprehensive system-level responses to IPV. For instance, Kaiser Permanente implemented these secondary and tertiary prevention strategies together with ongoing brief IPV training for medical providers and partnerships with community advocacy services [49]. Their comprehensive approach found a steady impact on IPV disclosures over time, with an 8-fold increase in IPV disclosures from 2000 to 2013 [50,51]. Similarly, the Veterans Health Administration has also implemented a national IPV response known as the Intimate Partner Violence Assistance Program [52]. This program oversees the implementation of annual IPV screening within PC and mental health clinics, disseminating evidence-based tertiary prevention interventions such as Recovering from IPV through Strengths and Empowerment (RISE) [53,54] and Strength at Home [55], and hiring coordinators at each site to assist with care coordination and to support educational efforts with healthcare staff.

## 4. Issues with the Current Healthcare Response to IPV

Despite the benefits of these current U.S. healthcare responses to address IPV and the potential reach of IPV screening, widespread barriers remain that prevent primary, secondary, and tertiary prevention strategies from reaching their full potential. For instance, at the system level, primary prevention efforts appear to be lacking at many large healthcare organizations [51]. Further, despite the benefits of screening for IPV in PC, implementation of traditional screening can be challenging. Screening is often limited due to structural factors such as lack of inadequate systems-level support (i.e., absence of adequate training, policies, protocols, and onsite clinical experts), competing priorities, and lack of time with patients [56,57]. Provider factors include personal discomfort with the topic, limited knowledge and misconceptions about IPV, and hesitancy in opening “Pandora’s Box” [58] because of the uncertainty of how to respond [56,59,60,61]. In addition, patient-level dynamics can also interfere with the successful implementation of screening. Patients might not disclose IPV due to the negative emotional effects of repeated violence and abuse, such as feelings of shame and guilt [60,62]. Additionally, patients may minimize or normalize aggression, abuse, violence and misreport on a screening measure because these behaviors have been normalized through past exposure [63].

Even if the above concerns were addressed, current IPV screening primarily emphasizes IPV experience and may not reliably identify all typologies of IPV, such as those who might also use IPV or respond to IPV from their partners with aggressive behaviors (i.e., bi-directional IPV) [46,64]. This is a critical concern because verbal, emotional, and psychological aggression is common and often bi-directional [10] and may not be recognized as problematic, unhealthy, or abusive in relationships [63]. IPV often starts with unhealthy relationship dynamics and relationship dissatisfaction that can escalate over time [65]. IPV screening has typically not focused on identifying these less severe, but still unhealthy, behaviors. While research indicated the negative consequences of IPV are greater for women and the current focus of IPV guidelines only address women, it is important to acknowledge this is not due to the absence of IPV among men [10] and non-binary individuals but because the research to date has focused primarily on younger (cis) gender heterosexual women. Although efforts are underway to fill in these research gaps, the current state of the field means that even under perfect administration conditions, IPV screening would still miss earlier stages of IPV, miss the context of IPV, and provide less information on underserved populations.

For patients that are identified through screening, an additional problem emerges when identifying the next steps. As noted above, IPV is typically heterogeneous, with behaviors ranging in frequency, intensity, and danger. Therefore, a patient with a positive screen may be responding based on the behavior on a continuum from unhealthy (i.e., less severe emotional or psychological abuse and maladaptive verbal and non-verbal communication) to overtly dangerous (i.e., more controlling psychological abuse and severe forms of physical or sexual violence). Furthermore, there may be a larger context around the IPV experience that is misunderstood. For example, a patient that systematically uses IPV to control their partner but occasionally experiences minor IPV as part of their partner’s self-defense would appear to be the victim in an experience-only screen. It is difficult for doctors or nurses to identify these nuances within the context of a brief medical evaluation, which in turn could lead to inaccurate recommendations.

If appropriate treatments are identified, implementing secondary and tertiary prevention is further challenged by the underutilization of IPV interventions due to multifaceted patient, provider, and system factors. Specifically, limited engagement with IPV interventions may be connected to how IPV screening efforts are implemented and sustained over time, the availability of IPV resources on site or in the community, how the healthcare provider responds to a disclosure (i.e., with indifference or judgment versus empathy), and patients’ overall readiness to seek to follow up [45,66,67]. This suggests passive referral to IPV interventions after screening may not be sufficient to initiate help-seeking and fully engage patients with IPV interventions. Randomized controlled trials of IPV screening have reported limited engagement with follow-up interventions. For example, Klevens et al. [68] found that 72% of women remembered receiving a list of resources at the one-year follow-up, and 4% reported contacting IPV resources, which was not significantly different from the control group. Similarly, MacMillan et al. [41] found that less than half of screened women reported having a discussion of IPV with their medical provider, and only 13% of women who were screened contacted IPV-focused resources. Groups receiving recommendations after screening did not contact resources at a higher rate than the control group. In their review of clinical IPV screening programs, Miller et al. [45] found a median of 32% of individuals screened received a referral to IPV interventions; of them, a median of 54% accessed these services. The authors found that successful follow-up and engagement with IPV interventions were influenced, in part, by the availability of onsite IPV interventions. Further, while access to onsite trauma-informed IPV resources is important, limited follow-up may also be influenced by patients’ lack of understanding of the purpose and scope of IPV interventions, trust in providers assuring confidentiality, or general readiness to engage [69]. In a primary care-based intervention in Australia, Hegarty and colleagues offered a different approach by training medical providers on how to deliver a 1–6 session brief counseling intervention based on Motivational Interviewing and problem-solving. When compared to the usual care group, 49% of women offered the brief counseling intervention attended a median of one session and saw no improvements in safety plans or quality of life, but did see improvements in depression at the 12-month follow-up. These improvements in depression were not sustained at the 24-month follow-up, where no group differences were found across all outcomes [70,71]. While essential resources, current secondary and tertiary initiatives face barriers to implementation and limitations in terms of their reach, as evidenced by mixed rates of disclosure, engagement with resources, provider and patient beliefs, and access to onsite resources. Further, these efforts have not been sensitive to identifying unhealthy or bi-directional relationship distress and aggression.

The U.S. Centers for Disease Control and Injury Prevention (CDC) encourages a multi-level, multi-sector approach to preventing IPV with an emphasis on primary prevention [29]. Therefore, we posit that expanding primary prevention efforts within health systems may address a critical gap in care, and create an opportunity to intervene early in the continuum of poor relationships, potentially halting the progression to IPV.

## 5. Leveraging Integrated Primary Care

New models of care delivery, such as integrated PC, offer opportunities to address current barriers and optimize IPV prevention. Integrated PC is a coordinated, person-centered, multidisciplinary approach to care that involves embedding a behavioral health provider into PC teams to assist in the delivery of comprehensive whole health services [72]. Models of integrated PC have been shown to reduce logistical and stigma-related barriers [73], yield high patient satisfaction and patient and provider acceptability [74,75], and improve mental healthcare utilization, including shorter wait times for care and increased likelihood of initiating and engaging in care [76]. As a result, integrated PC has seen substantial growth and has been implemented into numerous healthcare systems across the U.S. [72].

The increased growth in integrated PC can be leveraged to not only optimize the current secondary and tertiary prevention approaches within the healthcare system but also to innovate and build upon those efforts by supporting primary prevention efforts that could achieve a broader reach.

### 5.1. Optimizing Existing Secondary/Tertiary Prevention Approaches

The presence of a behavioral health provider within the PC team provides an opportunity to have an individual with the requisite training and skills to champion IPV efforts, facilitate patients in potential referrals, consult with providers, facilitate patient referrals, and provide brief treatments [77]. These are critical ingredients for the successful implementation of IPV screening programs in PC [56]. Specifically, the behavioral health provider can assist the PC team in optimizing their approach to discussions with patients about IPV screening and/or referrals through education as well as increase attention to how the team approaches screenings and referrals. For instance, passive didactic education is limited [78,79], but by embracing the interdisciplinary team created through embedding the behavioral health providers in PC, these providers can utilize experiential learning. This learning method is effective [80] and can allow for more applied learning about high-risk patient populations (e.g., same-sex couples) and discussions of IPV, health, and intervention resources. Furthermore, these behavioral health providers can also provide critical clinical services related to IPV, including nuanced assessment into the context of IPV and safety planning that go beyond the training and roles of medical providers. Additionally, they can be trained in effective, brief behavioral health interventions (such as Motivational Interviewing, cognitive behavioral therapy, or RISE [53]) that they can be offered in the PC setting [81]. Ease of access to these interventions, including the ability to receive IPV treatment within the convenience, safety, and privacy of the PC context, is desirable to individuals who experience IPV [82] and may enhance patients’ likelihood of following up on referrals and completing IPV interventions. This is key because such interventions can reduce psychological distress and enhance safety among individuals experiencing IPV [34,83,84].

Early research within integrated PC suggests there is potential to increase the likelihood of healthcare utilization for patients with IPV. Results of a study based in England provide some indication that this additional provider in the PC setting can assist in yielding positive results. Feder and colleagues [85] utilized a comprehensive training program, which included education on best practices for the identification of IPV and how to support the disclosure of IPV and referral to IPV resources. There was a 3-fold increase in disclosures of IPV among patients and a 22-fold increase in referrals to the IPV advocate relative to the control clinics. Central to this intervention was the availability of an onsite champion and a direct referral to an IPV advocate who provided follow-up services [85,86].

### 5.2. Initiating Universal Primary Prevention by Focusing on Relationship Health

While necessary, secondary and tertiary responses alone may not reduce IPV incidence due to their focus on downstream effects. Shifting the approach toward primary prevention is an essential next step toward improving patient outcomes. Given the importance relationship satisfaction has to overall health and happiness, integrated PC teams have the opportunity to supplement current IPV screening initiatives with new primary prevention strategies that offer a more holistic approach to addressing relationship concerns rather than only focusing on the most problematic relationships with detectable IPV. Within healthcare, primary prevention strategies often entail providing universal education to reach broad audiences. Such education shifts attention away from problem-based thinking to a population health framework by discussing the broader importance of healthy, satisfying relationships to one’s physical health. Recent research suggests that primary care populations express a strong interest in learning about links between their relationships, their current physical health, and their risk for future health problems [87]. This puts integrated PC providers in a unique position to discuss relationship health. Universal education helps overcome the limitations and barriers to IPV screening by providing knowledge about intimate relationships, what unhealthy, abusive, or violent relationships are, and how they influence health while also providing confidential resources. Inquiring about intimate relationships and, regardless of IPV disclosure, providing universal education about safe and healthy relationships broadens the focus from screening and disclosure-driven practices and interventions to a focus on education about healthy relationships and health promotion strategies. Such an approach to preventing IPV has preliminary acceptability and feasibility support from patients and providers [88]. One promising universal education approach that could be adapted for integrated PC is CUES: Confidentiality, Universal Education and Empowerment, Support). This model has been implemented and evaluated in reproductive and adolescent healthcare settings in the context of a particular type of IPV, reproductive coercion. CUES has demonstrated effectiveness in increasing patients’ knowledge of resources and use of safety and harm reduction strategies, as well as reducing reproductive coercion and IPV among adolescents and young women [89,90].

Whereas current screening guidelines tend to focus on women of childbearing age, a universal primary prevention approach to relationship health would entail discussing healthy relationships with all patients, including male and non-binary patients who may be missed by current guidelines. Together, this can normalize the discussion about IPV and motivate help-seeking behaviors [91]. This shifts the focus and goal of addressing the interaction between PC and patients from disclosure of IPV to one of building a holistic relationship focused on addressing the whole person [90]. Efforts focusing on increasing general IPV knowledge broadly have been effective in community-based and healthcare settings [89,92,93]. Importantly, such an initiative does not need to put an undue burden on a healthcare system or individual provider. In practice, this education may be distributed via standardized written educational materials, and/or a brief verbal communication.

## 6. Conclusions

Given the pronounced impact of IPV on patient well-being as well as the high demands on the healthcare system, it is encouraging that many healthcare organizations have taken steps to begin to identify and address IPV. However, IPV often entails a complex evolution and can transcend phases of life; thus, more work to identify comprehensive approaches to addressing IPV is indicated. Current secondary and tertiary strategies for addressing IPV are important but alone are not sufficient to fully prevent or intervene. While primary prevention strategies have not been fully developed and tested, they hold great potential for innovation in reaching a greater population of individuals and, thus, subsequently preventing IPV. Integrated PC is a particularly promising context for developing and testing such prevention strategies, which can build off the existing strengths of integrated PC. Behavioral health providers in primary care currently identify and intervene in a range of concerns highly correlated with IPV, such as depression, anxiety, and substance misuse. Thus, there is a unique opportunity to leverage this model of care to broaden the continuum of healthcare resources to those experiencing IPV while also potentially strengthening current secondary and tertiary efforts to identify and reduce IPV and promote recovery. Developing healthy relationships and communication can potentially reduce IPV and interrupt the negative effects on individuals, families, and communities [29]. The addition of a brief population health educational intervention could begin the process of a more effective, stepped approach to addressing IPV in healthcare settings.

## Figures and Tables

**Table 1:** Summary of Prevention.

	Primary Prevention	Secondary Prevention	Tertiary Prevention
Formal Definition			
Target Population	Population-level (prior to disorder onset)	Those with early-stage issues (prior to onset of harm)	Those who have already been harmed by a disorder
Goal	Prevent disorder	Prevent impact of disorder	Stop/mitigate further harm
Activities at Level	Health promotion and Specific protection	Early Diagnosis and Prompt Treatment	Disability Limitation and Rehabilitation
Relevant Guidance for IPV			
U.S.	2017 CDC Technical Package on Preventing IPV [29]	2018 USPTF Screening Recommendation [30]	1994 Violence Against Women Act [31]
Examples of IPV Responses to Healthcare System Response	CUES: Confidentiality, Universal Education and Empowerment, Support	IPV screening	RISE: Recovering from IPV through Strengths and Empowerment

Note. There are also international organizations that provide guidance, such as 2019 WHO RESPECT Women [32].

## Data Availability

No new data were created or analyzed in this study. Data sharing is not applicable to this article.
